# Supplementary material for: The Olfr151 Odorant Receptor Gene is Resistant to Activation in Embryonic Stem Cells
Source: bioRxiv. 2026 Feb 20:2026.02.19.706738. Preprint. [Version 1] doi: 10.64898/2026.02.19.706738 (PMC12934667; doi:10.64898/2026.02.19.706738)
Supplement: 1 [file NIHPP2026.02.19.706738v1-supplement-1.pdf]

bioRxiv preprint doi: https://doi.org/10.64898/2026.02.19.706738; this version posted February 20, 2026. The copyright holder for this preprint (which was not certified by peer review) is the author/funder, who has granted bioRxiv a license to display the preprint in perpetuity. It is made available under aCC-BY 4.0 International license.

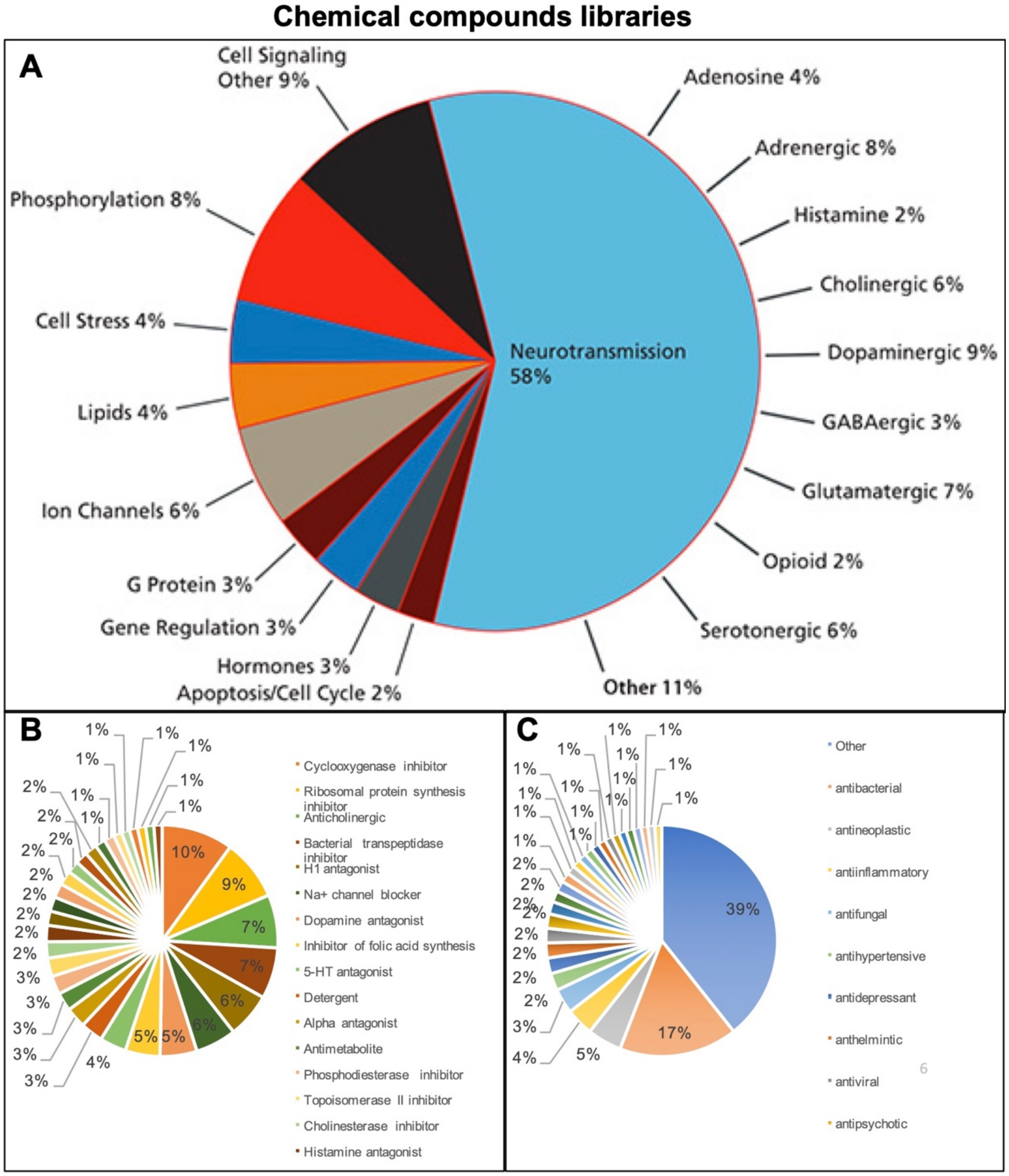

Supplementary Fig. S1. Chemical compounds libraries.
(A) 1280 LOPAC chemical compounds are grouped together based on the mechanisms of their activities (LOPAC®1280). **(B)** Prestwick chemical compounds are grouped based on their chemical activity (Prestwick Chemical). **(C)** MicroSource chemical compounds are grouped based on similar mechanisms of activity upon the cells (MicroSource Discovery Systems, Inc.).

bioRxiv preprint doi: https://doi.org/10.64898/2026.02.19.706738; this version posted February 20, 2026. The copyright holder for this preprint (which was not certified by peer review) is the author/funder, who has granted bioRxiv a license to display the preprint in perpetuity. It is made available under aCC-BY 4.0 International license.

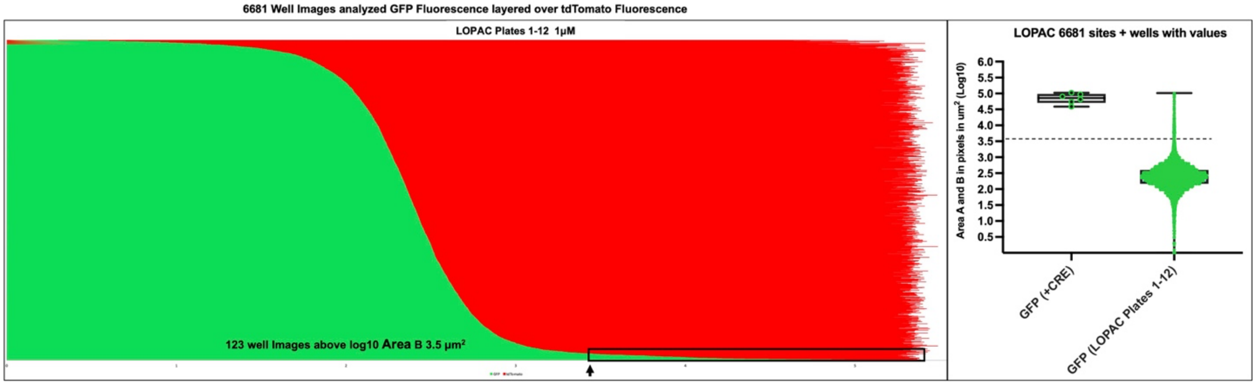

Supplementary Fig. S2. Fluorescent expression in mESCs as a result of LOPAC chemical compounds treatments.

All LOPAC treatments (12x 384-well plates, in two concentrations) yielded 6881 images (A combination of nine and four images per well) log10 Area (fluorescence area, μm^2) of tdTomato (red) and GFP (green) fluorescence in reporter cells. The green and red log10 Area values were ranked based on increasing green fluorescence (log10 Area B, μm^2). 123 images contained GFP fluorescence shown within black rectangle above the threshold of log10 Area B = 3.5μm^2 (see Fig. 4).

bioRxiv preprint doi: https://doi.org/10.64898/2026.02.19.706738; this version posted February 20, 2026. The copyright holder for this preprint (which was not certified by peer review) is the author/funder, who has granted bioRxiv a license to display the preprint in perpetuity. It is made available under aCC-BY 4.0 International license.

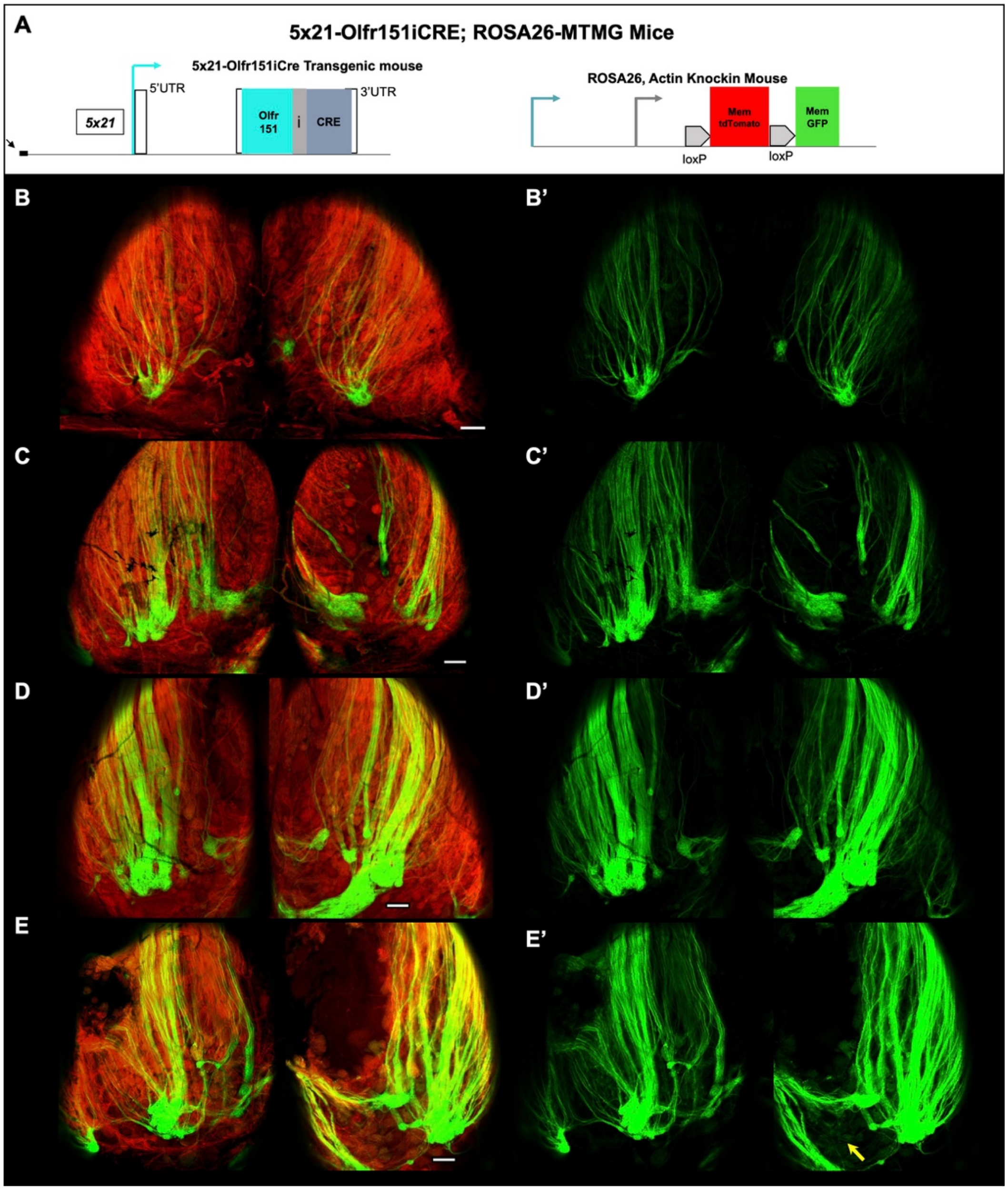

Supplementary Fig. S3. Four 5x21-Olfr151iCRE transgenes in the ROSA26-MTMG reporter mouse.
(A-D) Overlays of wholemount tdTomato and GFP fluorescent microscopy of olfactory bulbs. (A) Olfr151iCRE transgene mice, containing 5x21 enhancer, were generated and crossed with ROSA26-MTMG mice. (B) 5x21-Olfr151iCre strain β (beta) line. (C) 5x21-Olfr151iCre strain ɣ (delta). (D) 5x21-Olfr151iCre strain ɣ (gamma). (E) 5x21-Olfr151iCre strain α (alpha). (B-E) All four transgenic lines contained coalesced GFP labeled axons into glomeruli that were larger than those found in the Olfr151iCRE knockin mice (Fig. 1B), which is due to the coalescence of greater number of Olfr151 axons. Scale bar = 200µm. **(A'-D')** Wholemount GFP fluorescent microscopy of olfactory bulbs from (A-D).
